# Reduced Dietary Sodium Intake Increases Heart Rate. A Meta-Analysis of 63 Randomized Controlled Trials Including 72 Study Populations

**DOI:** 10.3389/fphys.2016.00111

**Published:** 2016-03-24

**Authors:** Niels A. Graudal, Thorbjørn Hubeck-Graudal, Gesche Jürgens

**Affiliations:** ^1^Department VRR4242, Rigshospitalet, Copenhagen University HospitalCopenhagen, Denmark; ^2^Department of Nuclear Medicine, Herning HospitalHerning, Denmark; ^3^Clinical Pharmacological Unit, Zealand University HospitalRoskilde, Denmark

**Keywords:** dietary sodium, heart rate, blood pressure, side-effect, meta-analysis

## Abstract

Reduced dietary sodium intake (sodium reduction) increases heart rate in some studies of animals and humans. As heart rate is independently associated with the development of heart failure and increased risk of premature death a potential increase in heart rate could be a harmful side-effect of sodium reduction. The purpose of the present meta-analysis was to investigate the effect of sodium reduction on heart rate. Relevant studies were retrieved from an updated pool of 176 randomized controlled trials (RCTs) published in the period 1973–2014. Sixty-three of the RCTs including 72 study populations reported data on heart rate. In a meta-analysis of these data sodium reduction increased heart rate with 1.65 beats per minute [95% CI: 1.19, 2.11], *p* < 0.00001, corresponding to 2.4% of the baseline heart rate. This effect was independent of baseline blood pressure. In conclusion sodium reduction increases heart rate by as much (2.4%) as it decreases blood pressure (2.5%). This side-effect, which may cause harmful health effects, contributes to the need for a revision of the present dietary guidelines.

## Introduction

About 0.5 g salt per day is sufficient to maintain vital physiological effects, but a daily habitual salt intake up to 40 g has been described (Battarbee, 1978). Sixty-five percent of the World's populations eat salt in the interval 8–10 g and 95% in the interval 6–12 g (McCarron et al., 2013). Consequently, world-wide the salt intake is very tightly regulated (Geerling and Loewy, 2008) in the low end of the tolerable interval of 0.5–40 g. Still, some health institutions consider this salt intake to be unhealthy due to an assumed association of sodium with blood pressure (BP) (WHO, 2012; US Department of Health and Human Services, 2015), and therefore recommend lowering sodium intake below 100 mmol (2300 mg, 5.8 g salt) (Box 1). However, meta-analyses question this assumption, as the effect of sodium reduction (SR) on BP is only 1.27/0.05 mmHg in individuals with a normal BP (Graudal et al., 2011) without indication of a dose-response relationship (Graudal et al., 2015). A moderate effect in individuals with hypertension (5.5/2.75 mmHg) (Graudal et al., 2011) does not justify SR for the whole population.

Box 1 1000 mg of sodium (Na) corresponds to 2542 mg salt (NaCl) (43 mmol). 100 mmol NaCl corresponds to 2299 mg of sodium and 5844 mg of salt. In this article we use the term “sodium” and the unit “mmol.”

Compensating physiological mechanisms, like increases in renin and aldosterone (Brunner et al., 1972; Graudal et al., 1998) and noradrenalin and adrenalin (Graudal et al., 1998, 2011) during reduced sodium intake may contribute not only to maintain BP, but also to increase heart rate (HR). Animal experiments have shown that sodium reduction to one tenth of normal intake increase HR with 25% in rats (Ely et al., 1985). Folkow et al. concluded that “when it comes to the potential impact of the salt intake level which, when altered, in most subjects change BP and HR in *opposite* directions, these two parameters should be carefully and jointly analyzed” and “in studies of the effects of salt intake on blood pressure, influences on heart rate are usually neglected even though the long-term load on both left ventricle and systemic arteries is better related to the product of HR × BP than to pressure alone” (Folkow and Ely, 1998). This indication of the clinical importance of HR is further emphasized by prospective observational studies, which have shown that HR is independently associated with mortality (Jensen et al., 2012; Ho et al., 2014), just as BP (Lewington et al., 2002). Such an effect on HR could contribute to the finding of an association of low sodium intake with increased mortality in several population studies (Graudal et al., 2014; O'Donnell et al., 2014; Pfister et al., 2014).

The purpose of the present meta-analysis was to investigate the effect of SR on HR at different blood pressure levels. We also intended to identify longitudinal studies to define a time point for maximal efficacy of SR on HR and studies investigating different doses of sodium intake to establish a dose-response relationship between sodium intake and HR.

## Methods

### Trial search

Studies to be included were selected from an updated pool of trials identified in a previous review (Graudal et al., 2011), in which the details of the search procedure are described. Using this procedure the literature search was updated in April 2015.

### Types of studies

Only randomized controlled trials (RCTs) were included.

### Participants

Studies of healthy persons or persons with hypertension irrespective of age and race were included. Studies systematically investigating patients with diseases other than elevated BP (e.g., diabetes or heart failure), were excluded.

### Intervention

The intervention is reduced sodium chloride intake (sodium reduction: SR). Studies, in which reduced sodium intake was controlled by means of 24-h urine sodium excretion or 8-h urine sodium excretion, were included. Studies not designed to control for the confounding effect of co-interventions were excluded, i.e., studies treating persons with a concomitant intervention such as an antihypertensive medication, potassium supplementation or weight reduction were only included if the concomitant intervention was identical in both the reduced and the usual sodium diet groups.

### Primary outcome

Effect on HR calculated as the difference between the change during intervention in the low and usual sodium intake dietary groups. HR is measured as beats per minute (bpm) on the first and the last day of the intervention.

### Supplementary analysis

The effect of SR on HR in subgroups defined by the 25, 50, and 75% BP-distribution percentiles of the American population (Wright et al., 2011) were investigated.

### Secondary outcomes

The within study effect of SR on HR at different time points and at different doses of sodium reduction.

### Data extraction

Two authors independently recorded the following data from each trial: HR (SD) before and after intervention for each measurement time and for each dose of sodium reduction; the sample size (N); the mean age of participants; SR measured as the difference between 24-h urinary sodium excretion during low-sodium and usual-sodium diets and standard deviation (SD); SBP (SD) and DBP (SD) before and after intervention. If there were discrepancies between reviewers they looked at the data together and came to an agreement.

### Risk of bias (quality) assessment

This was performed using the Cochrane Risk of bias tool, including recording of allocation, blinding, incomplete outcome data, and selective reporting (Higgins and Green, 2011). Reporting bias was evaluated by an estimate of asymmetry in Funnel plots (Cochrane Collaboration, 2011; Higgins and Green, 2011).

### Heterogeneity

A chi-squared test included in the forest plot was used to assess whether observed differences in results are compatible with chance alone. A low *P* value (or a large chi-squared statistic relative to its degree of freedom) provides evidence of heterogeneity of intervention effects (variation in effect estimates beyond chance) (Higgins and Green, 2011).

### Data synthesis

The HR outcome was defined as the weighted mean difference (MD) between the changes from baseline to end of treatment during low and usual sodium diets. The combined effect measures were compared by means of the inverse variance method in Review Manager (Cochrane Collaboration, 2011). As we accumulated data from a series of studies that had been performed by researchers operating independently, and as the goal of the analysis was to extrapolate to other populations, we used a random effect model in our primary analysis to estimate the summary measure as the mean of a distribution of effects. In a secondary analysis we used a fixed-effect model assuming that the true effect size for all studies is identical. As we move from random effect to fixed effect, extreme studies will gain influence if they are large, and will lose influence if they are small. If there is no heterogeneity (tau^2^ = 0 and *I*^2^ = 0), the two models are identical.

## Results

### Trial identification

**Figure 1:**
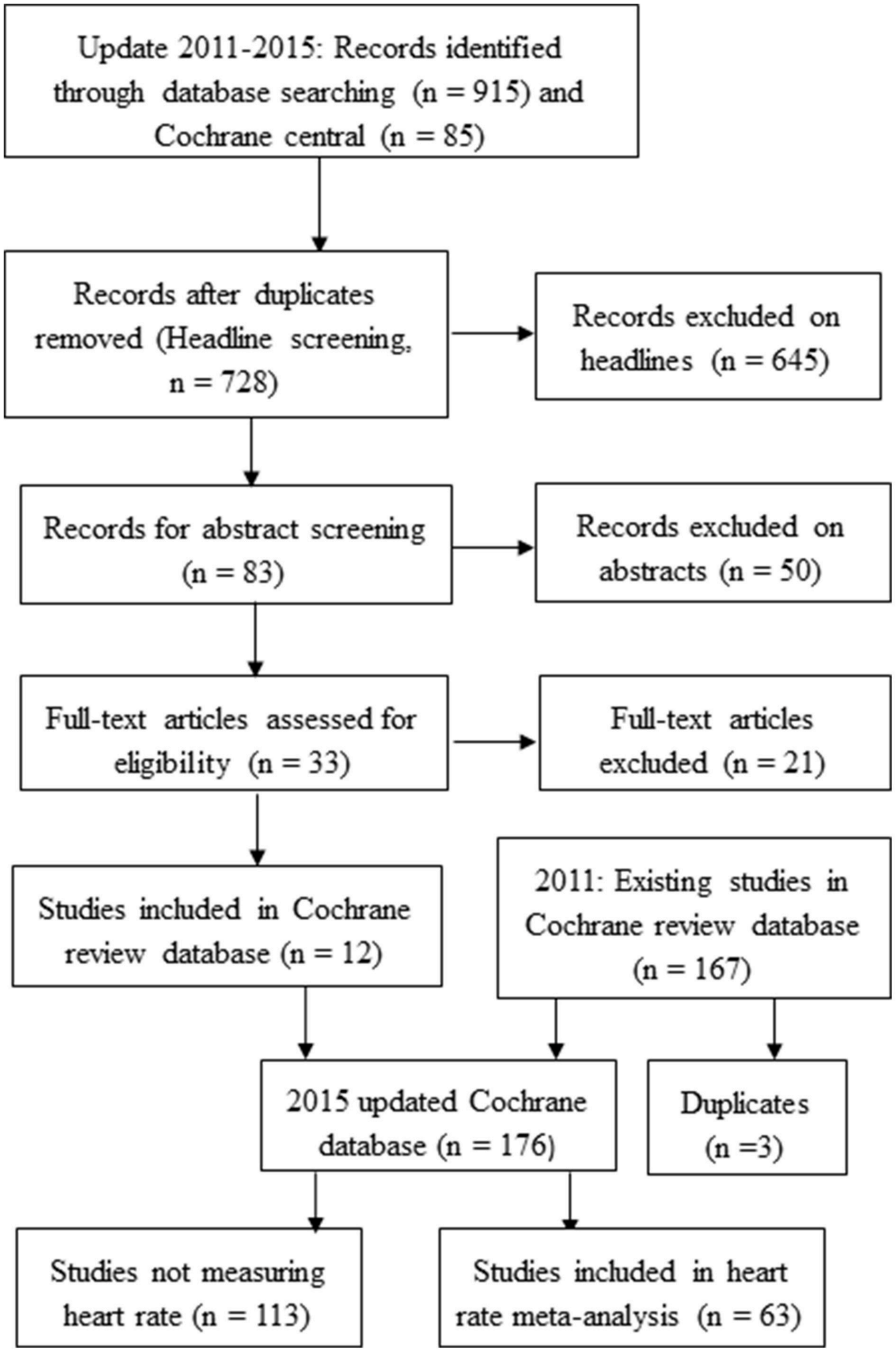
**Flow diagram of the trial identification**.

The last update of the 2011 search was performed April 15 2015. During the update the de-duplicated results from the searches revealed 728 articles. On the basis of headlines 645 were excluded. Eighty-three abstracts were read and 33 full articles obtained of which 12 fulfilled the inclusion criteria. During the process we identified 3 duplicates in the 2011 review (Graudal et al., 2011), which were eliminated. A total of 176 references (164 from the 2011 review plus 12 new references) were thus included in the present updated version. These 176 references included 198 study populations. Information on HR was available in 72 of these study populations, described in 63 references. Figure 1 shows a flow diagram of the trial identification.

### Trial characteristics

The 72 study populations included in the present meta-analysis are shown in Table 1.

**Table 1:** **Study characteristics and references of included studies**.

**No**.	**Study ID**	**Dur. Days**	***N***	**Age (years)**	**Sodium reduction mmol**	**HR (beats/min)**	**References**
1	Paulsen	4	22	24	47	54.0	Scand J Clin Lab Invest 2009;69:323–9
2	Seals	90	35	63.5	46	68.0	J Am Coll Cardiol 2001;38:506–13
3	Morgan	14	16	63	50	77.0	J Hum Hypertens 1988;1:311–5
4	Grobee	42	40	24	72	70.0	BMJ 1987;293:27–9
5	Dickinson	42	25	35.1	42	68.0	Atherosclerosis 2014;232:211–6
6	Ishimitsu N	7	7	53	195	64.0	Clin Sci 1996;91:293–8
7	Fotherby	35	17	73	79	75.0	J Hypertens 1993;11:657–63
8	He B	42	69	50	44	68.0	Hypertension 2009;54:482–8
9	ANHMRCDSSMC	48	103	58.4	63	75.7	Lancet 1989;1:399–402
10	Doig	4	8	25	117		J Cardiovasc Pharm 1995;25:511–17
11	Sharma	6	23	25	246	64.3	J Hypertens 1991;9:329–35
12	Gillies	42	24	56.7	77	67.2	Clin Exp Pharm Phys 1984;11:395–8
13	Starmans-Kool	14	10	32	97	56.0	J Appl Physiol 2011;110:468–71
14	Jessani	7	184	49.5	81	82.0	Am J Hypertens 2008;21:1238–1244
15	Beeks	7	117	53.4	99	63.5	Hypertension 2004;44:419–23
16	Wing	42	17	61	59	80.0	Blood Pressure 1998;7:299–307
17	Schorr	28	16	64.1	61	78.0	J Hypertens 1996;14:131–5
18	Fliser	7	7	25.5	180	59.0	Eur J Clin Invest 1995;25:39–43
19	Bruun H	4	12	47	331	73.0	J Hypertens 1990;8:219–27
20	Chiolero H	7	38	43	183	76.0	J Hypertens 2000;36:631–7
21	Benetos	28	20	41.5	78	75.3	J Hypertens 1992;10:355–60
22	Skrabal	14	52	23	156	69.3	Hypertension 1984;6:152–8
23	Ferri	14	61	47.1	264	73.2	J Am Soc Nephrol 1996;7:443–53
24	Sharma	7	40	25	214	61.0	Hypertension 1990;16:407–13
25	Davrath	5	8	25.1	96	63.5	Aviat Space Env Med 1999;70:577–82
26	Miller	14	36	23.4	58	60.0	Psychosom Med 1995;57:381–9
27	Ishimitsu H	7	23	55	193	59.7	Clin Sci 1996;91:293–8
28	Cooper	24	113	16.3	64	81.0	J Hypertens 1984;2:361–6
29	Bonfils N	5	12	39	140	64.0	J Hypertens 2013;31:2220–9
30	Graffe	4	21	26	172	65.0	Am J Physiol Renal Physiol 2012;15:F264–75
31	Tzemos	5	16	27	149	67.0	Hypertension 2008;51:1525–35
32	Dishy	6	25	34	300	67.0	J Hypertens 2003;21:153–7
33	Zanchi	7	9	25	250	66.0	J Clin Endocrin Metab 2004;89:1140–5
34	Burnier	7	15	22.7	188	71.0	J Hypertens 2000;18:1657–64
35	Bech	5	12	23.8	235	56.0	Am J Physiol 1998;274:914–23
36	Herlitz	4	6	46	98	73.0	Blood Press 1998;7:47–52
37	He W	42	71	52	55	65.0	Hypertension 2009;54:482–8
38	Hyperpath C2	7	211	49.2	211	63.5	Hypertension 2012;60:1359–66
39	Draaijer	7	10	41	259	69.8	J Hum Hypertens 1995;9:263–9
40	Schorr	7	187	25	206	56.6	J Hypertens 1999;17:475–9
41	Ambrosioni	42	25	23	60	76.0	Hypertension 1982;4:789–94
42	He A	42	29	47	68	67.0	Hypertension 2009;54:482–8
43	Chiolero N	7	12	40	201	67.0	J Hypertens 2000;36:631–7
44	Sullivan N	4	27	28.8	146	61.0	Hypertension 1980;2:506–14
45	Fuchs	9	17	20	229	64.7	Brazi J Med Biol Res 1987;20:25–34
46	Sullivan H	4	19	27	153	64.0	Hypertension 1980;2:506–14
47	Johnson	14	40	68.8	73		J Hypertens 2001;19:1053–60
48	Cuzzola	14	19	47	161	71.0	Am J Hypertens 2001;14:224–30
49	Stein	5	7	33.7	183	61.2	Clin Pharm Ther 1995;58:425–33
50	Parker	28	59	52	73	74.0	Hypertension 1990;16:398–406
51	Uzu	7	70	50.5	173	64.8	Am J Hypertens 1999;12:35–9
52	Mallamaci	14	32	48	165	70.0	J Hypertens 2013;31:1424–30
53	Bonfils OW	5	12	39	157	68.0	J Hypertens 2013;31:2220–9
54	Damgaard	7	12	56.8	129	64.0	Am J Physiol - Reg Integ Comp Physiol 2006;290:R1294-R1301
55	Bruun N	4	10	46	341	60.0	J Hypertens 1990;8:219–27
56	Mark	10	6	28	305	63.3	Circ Res 1975;(Suppl 1):36–7: I194–I198
57	Overlack	7	46	45.3	245	64.7	Am J Hypertens 1995;8:829–36
58	Ruppert	7	163	38	274	63.2	Hypertens 1993;11:743–9
59	Fliser	16	16	25	187	77.1	J Hypertens 1993;6:320–4
60	Sciarrone	56	91	53.5	82		J Hypertens 1992;10:287–98
61	Allen	5	70	24	83	72.0	J Hypertens 2014;32:374–82
62	Mak	7	13	24	190	56.0	Eur Heart Journal Cardiovascular Imaging 2013;14:1092–8
63	Inoue	7	14	46	293	71.5	J Hum Hypertens 1996;10:523–9
64	Townsend	6	20	30	171	67.0	Clin Sci (London) 2007;113:141–8
65	Boero	14	13	51	209	76.0	Min Urol Nefrol 2000;52:13–6
66	Hargreaves	14	8	23.4	106	59.0	Clin Sci 1989;76:553–7
67	Skrabal	14	20	23	150	62.2	Lancet 1981; II:895–900
68	Carey C1	7	185	47	203	68.1	Hypertension 2012;60:1359–66
69	Bonfils H	5	12	43	131	73.0	J Hypertens 2013;31:2220–9
70	Sudhir	12	6	34.7	133	61.0	Clin Sci 1989;77:605–10
71	Melander	28	39	53	89	67.7	J Hypertens 2007;25:619–27
72	Friberg	13	10	33.3	117	63.0	Hypertension 1990;16:121–30

### Effect of SR on HR

**Figure 2:**
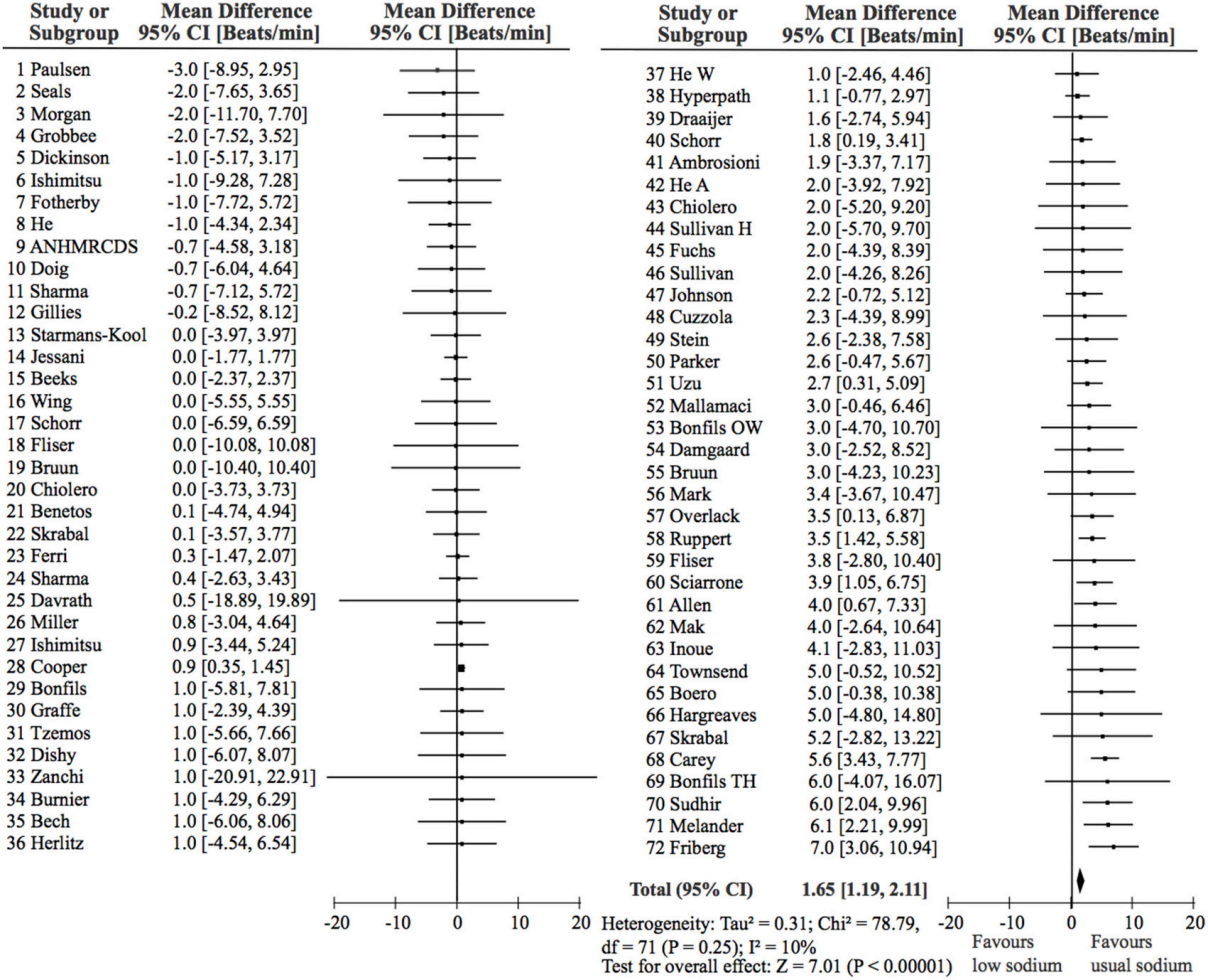
**Forrest plot showing effect of sodium reduction on heart rate in 72 study populations**. The overall effect of sodium reduction is 1.65 b.p.m. The studies are ordered according to the effect size and correspond to the order in Table 1.

Figure 2 shows that the effect of SR is highly significant, although clinically small: 1.65 bpm [95%CI: 1.19, 2.11], *p* < 0.00001, random effect, or 1.40 bpm [95% CI: 1.05, 1.75], fixed effect. As the mean baseline HR for the whole population was 67.8 bpm, this corresponds to an effect of 2.4% (1.65/67.8). Table 2 shows the effect of SR on HR in BP-distribution quartiles defined by quartiles of the American population (Wright et al., 2011). The effect of SR on HR was significant in all four quartiles indicating that the effect of SR on HR is independent of the baseline BP.

**Table 2:** **Mean heart rate effect of sodium reduction stratified by blood pressure quartiles of the American population**.

**Systolic BP percentile, mmHg**	**Number of studies (participants)**	**Heart rate, MD (95%CI) (beats/min)**	**Z (p)**
0–25 %, −110	3 (304)	0.91 [0.37, 1.45]	3.28 (0.001)
25–50 %, 110–119	18 (1204)	2.18 [1.26, 3.11]	4.62 (0.00001)
50–75 %, 119–131	20 (1472)	1.99 [0.72, 3.27]	3.06 (0.002)
75–100 %, 131 –	31 (2452)	1.41 [0.75, 2.07]	4.21 (0.0001)

The most contrasting risk of bias element was blinding (24 double-blind vs. 48 open studies). In 24 double-blind studies (*n* = 1386) the effect of SR was 1.34 bpm [0.54, 2.15], *p* < 0.001, random and fixed effect (*I*^2^ = 0%). In 48 open studies (*n* = 4040) the effect of SR was 1.83 bpm [1.24, 2.43], *p* < 0.00001, random effect, or 1.42 bpm [1.03, 1.81], *p* < 0.00001, fixed effect (*I*^2^ = 18%).

### Longitudinal effect of SR on HR

Only 2 studies gave such results. In the study by Ruppert et al. (1993) the effect of SR on HR was 1.6 bpm at week 1 and 1.1 bpm at week 4. In the study by Fuchs et al. (1987) the outcome varied at 3, 6, and 9 days but without an obvious trend (Table 3).

**Table 3:** **Changes in mean heart rate in studies investigating different doses of sodium**.

**References**	**Minor sodium reduction (b/m)**	**Moderate sodium reduction**	**Extreme sodium reduction**	**Study Duration, days**
45 Fuchs (1987)	NA	4.6	3.3	3
45 Fuchs (1987)	NA	1.5	1.5	6
45 Fuchs (1987)	NA	−0.9	4.5	9
19 Bruun (1990) (N)	NA	1	2	4
19 Bruun (1990) (H)	NA	0	1	4
34 Burnier (2000)	NA	1	2	7
47 Johnson (2001)	−1	1.2	1.8	14

### Dose-response relationship of SR vs. HR

Table 3 shows the outcome of seven dose-response analyses in four RCTs (Fuchs et al., 1987; Bruun et al., 1990; Burnier et al., 2000; Johnson et al., 2001). The trend is that HR increases with increasing SR but the number of investigations is few and the differences are small.

## Discussion

This meta-analysis shows that SR increases HR. The increase is statistically highly significant (*Z* = 7.0, *p* < 0.00001), but the clinical significance of the size of the increase, which is 1.65 bpm corresponding to 2.4%, is debatable. The effect size matches quantitatively the effect of SR on SBP in the investigated RCTs, which is 3.4 mmHg (mean baseline SBP = 135 mmHg), corresponding to 2.5% (3.4/135). However, in contrast to the BP effect, which is moderate in hypertensives and low in normotensives, the HR effect is independent of baseline BP. This means that individuals with a BP in the lower 50% percentile of the BP distribution may get an increase in HR without a reduction in BP. Study blinding had no significant influence on the effect size. Two longitudinal studies were too few to determine the time of maximal efficacy (Fuchs et al., 1987; Ruppert et al., 1993). Seven dose-response relationships from 4 RCTs (Fuchs et al., 1987; Bruun et al., 1990; Burnier et al., 2000; Johnson et al., 2001) indicate that a dose-response relationship does exist, but the data are not sufficient for a reliable conclusion. Previously we have shown that ethnicity only has a minor impact on BP (Graudal and Jürgens, 2015), but we could not determine whether ethnicity has an impact on the effect of SR on HR, as only 2 Black and 4 Asian study populations were identified in the present meta-analysis.

A series of population studies have not been able to demonstrate a beneficial association between low sodium intake and health outcomes. A recent IOM report [Institute of Medicine (IOM), 2013] concluded “Science was insufficient and inadequate to establish whether reducing sodium intake below 2300 mg/d (100 mmol) either decreases or increases CVD risk in the general population.” A later meta-analysis of these population studies found that a sodium intake below 115 mmol was associated with increased mortality, as was a sodium intake above 215 mmol (Graudal et al., 2014). This U-shaped relation between sodium intake and mortality has been identified in several individual population studies (Thomas et al., 2011; O'Donnell et al., 2014; Pfister et al., 2014). The reason for this U-shape could be that SR has side effects as indicated in our previous meta-analysis, which shows that SR significantly increases plasma renin, plasma aldosterone, plasma adrenaline, plasma noradrenaline, plasma cholesterol, and plasma triglyceride (Graudal et al., 2011). In addition, the present finding of an increase in HR can be considered a side-effect, because HR has been associated with mortality (Jensen et al., 2012; Ho et al., 2014) and development of heart insufficiency (Pfister et al., 2012) in observational studies. In the Copenhagen City Study a 10 bpm increase in resting HR was associated with increased cardiovascular and all-cause mortality in both univariate (about 20%) and multivariate (about 10%) models (Jensen et al., 2012), and in the Framingham study an 11 bpm increase in resting HR was also associated with increased cardiovascular (18%) and all-cause (17%) mortality (Ho et al., 2014). A 20 mmHg increase in SBP leads to about 100% increase in mortality (Lewington et al., 2002). Assuming that these associations are linear, it can be estimated that an increase in HR of about 3 bpm outbalances the effect of a reduction in SBP of 1 mmHg. As mentioned the clinical significance of a 2.4% increase in HR may be questionable. However, the SR recommendations of the health institutions are based on the assumption that a small BP reduction in the whole population will lead to a significant reduction in mortality (WHO, 2012; US Department of Health and Human Services, 2015). Assuming that this concept is correct a 2.4% increase in HR should have the opposite effect especially when combined with increases in other factors know to be predictive of mortality, such as renin, noradrenalin and cholesterol. Indeed, observational population studies indicate that side effects of SR trump the BP effect (Thomas et al., 2011; Graudal et al., 2014; O'Donnell et al., 2014; Pfister et al., 2014). This seems to be the case, not only in healthy normotensive individuals, but also in heart failure patients. In a recent study of symptomatic patients with chronic heart failure sodium restriction was associated with significantly higher risk of death or HF-hospitalization (hazard rate = 1.85; 95% confidence interval: 1.21 to 2.84; *p* < 0.004) (Doukky et al., 2016). These findings indicate that health institutions in their Dietary Guidelines (US Department of Health and Human Services, 2015; DeSalvo et al., 2016) to a greater extent should recognize the lack of RCTs on the effect of SR on health outcomes, the possible side effects of SR and the association of low sodium intake with increased mortality in observational studies (Graudal, 2016). In the light of the ongoing debate, the process of preparing guidelines should include “full transparency, a lack of bias, and the inclusion and consideration of all of the latest available research and scientific evidence, even that which challenges current dietary recommendations” (Whoriskey, 2016). The present sodium recommendations do not fulfill these criteria. In conclusion SR increases HR. This should be considered a side-effect, which may have a harmful effect on the general population. This effect contributes to the need for a revision of the present dietary guidelines, which now has been initiated by the American Congress (Whoriskey, 2016).

## Author contributions

NG: Substantial contributions to the conception, design of the work; and the acquisition, analysis, or interpretation of data for the work, Drafting the work, and Final approval of the version to be published. Agreement to be accountable for all aspects of the work in ensuring that questions related to the accuracy or integrity of any part of the work are appropriately investigated and resolved. TH: Substantial contributions to the acquisition, analysis, and interpretation of data for the work, Revising it critically for important intellectual content, and Final approval of the version to be published. Agreement to be accountable for all aspects of the work in ensuring that questions related to the accuracy or integrity of any part of the work are appropriately investigated and resolved. GJ: Substantial contributions to the acquisition, analysis, and interpretation of data for the work, Revising it critically for important intellectual content, and Final approval of the version to be published. Agreement to be accountable for all aspects of the work in ensuring that questions related to the accuracy or integrity of any part of the work are appropriately investigated and resolved.

### Conflict of interest statement

The authors declare that the research was conducted in the absence of any commercial or financial relationships that could be construed as a potential conflict of interest.
